# Integration of Preoperative Neutrophil-to-Lymphocyte Ratio into Machine Learning Models for Predicting Lymph Node Metastasis in CRC

**DOI:** 10.3390/cancers18152370

**Published:** 2026-07-23

**Authors:** Yaqi Zhang, Yujing He, Ziyu Wang, Yingke Sun, Haijie Zhi, Hongliang Wu, Bingtao Weng, Zhangfa Song

**Affiliations:** 1 Department of Colorectal Surgery, Sir Run Run Shaw Hospital, School of Medicine, Zhejiang University, Hangzhou 310016, China; 2 Zhejiang Key Laboratory of Biotherapy, Hangzhou 310016, China; 3 Key Laboratory of Integrated Traditional Chinese and Western Medicine Research on Anorectal Diseases of Zhejiang Province, Hangzhou 310016, China; 4 School of Life Sciences and Health Engineering, Jiangnan University, Wuxi 214122, China; 5 School of Basic Medical Sciences and Forensic Medicine, Hangzhou Medical College, Hangzhou 310007, China; 6 Institute of Advanced Equipment, College of Energy Engineering, Zhejiang University, Hangzhou 310007, China; 7 Department of Emergency Medicine, Sir Run Run Shaw Hospital, School of Medicine, Zhejiang University, Hangzhou 310016, China; 8 School of Public Health, School of Medicine, Zhejiang University, Hangzhou 310030, China

**Keywords:** colorectal cancer (CRC), lymph node metastasis (LNM), neutrophil-to-lymphocyte ratio (NLR), machine learning (ML)

## Abstract

**Background**: Accurate preoperative prediction of lymph node metastasis (LNM) is essential for tailoring treatment strategies in T1 colorectal cancer (CRC). Although the neutrophil-to-lymphocyte ratio (NLR), an easily acquired inflammatory biomarker, correlates with tumor progression, its incremental value when incorporated into machine learning (ML) models for predicting LNM in T1 CRC remains unclear. Therefore, this study evaluated the independent predictive value of preoperative NLR for LNM and constructed interpretative ML models to stratify LNM risk in T1 CRC. **Methods**: We retrospectively enrolled 533 pathologically confirmed T1 CRC patients. NLR was calculated as the neutrophil–lymphocyte count ratio. Multivariable logistic regression identified NLR’s independent correlation with LNM. Features were screened via combined least absolute shrinkage and selection operator (LASSO) regression and the Boruta algorithm. Six ML models, including Logistic Regression (LR), Random Forest (RF), XGBoost, LightGBM, CatBoost, and Logistic Regression with Splines (LR-Spline), were developed and validated. Model performance was evaluated in terms of discrimination, calibration, and clinical utility. Shapley additive explanations (SHAP) were applied for feature interpretability. **Results**: Elevated preoperative NLR was independently associated with an increased risk of LNM (adjusted OR = 2.83, 95% CI: 1.48–5.41, *p* = 0.002), exhibiting stability across all clinical and pathological subgroups. The optimized LightGBM outperformed other models with an area under the receiver operating characteristic curve (AUC) of 0.768, alongside favorable clinical utility. **Conclusions**: An elevated preoperative NLR is a robust, independent predictor of LNM in patients with T1 CRC. An optimized LightGBM model integrating NLR with routine clinicopathological indicators offers accurate risk stratification, potentially refining surgical decision-making and sparing low-risk T1 CRC patients from unnecessary radical resections.

## Simple Summary

In early-stage colorectal cancer, preoperative assessment of lymph node metastasis (LNM) remains challenging, often resulting in overtreatment with unnecessary radical surgery in low-risk patients. In this study, we investigated a routinely available systemic inflammatory marker and developed multiple machine learning-based predictive models using clinical data from 533 patients. Our results demonstrated that elevated levels of this inflammatory index were independently associated with an increased risk of LNM. Among the evaluated models, an optimized machine learning model achieved the best predictive performance. This model enables effective preoperative stratification of patients into high- and low-risk groups, thereby supporting individualized surgical decision-making and potentially reducing unnecessary extensive surgical procedures.

## 1. Introduction

Colorectal cancer (CRC) is globally recognized as the third most common primary malignancy and the second leading cause of cancer-related mortality, constituting a severe and growing global healthcare burden [1]. The widespread implementation of population-based CRC screening programs in recent years has markedly improved the early detection rate of T1-stage CRC [2], bringing this early-stage disease into the focus of routine clinical management. Currently, endoscopic resection (ER) serves as the primary standard treatment for T1 CRC. Compared with traditional radical surgery, ER features organ preservation and minimal invasiveness, offering superior quality of life for patients. However, the oncological safety of ER is heavily challenged by the potential for lymph node metastasis (LNM) [3]. While T1 CRC is generally associated with a favorable prognosis, approximately 10% of patients harbor occult LNM at the time of diagnosis [4]. The presence of LNM drastically reduces long-term survival and drives recurrence, making its accurate assessment the central oncological challenge in managing T1 CRC [5]. International clinical guidelines from Europe, the United States, and Japan recommend additional radical surgical resection with lymphadenectomy following ER based on high-risk pathological features, such as invasion depth, lymphovascular invasion (LVI), high histological grade, and tumor budding [6,7,8,9]. However, these criteria accurately predict positive lymph node status in only 8% to 16% of patients. Consequently, up to 90% of patients undergo unnecessary secondary radical surgeries [10]. Relying solely on these traditional histopathological parameters results in significant clinical overtreatment, highlighting an urgent need for novel, non-invasive, and easily accessible pre-therapeutic biomarkers to refine LNM risk stratification.

In recent years, the crucial role of the systemic inflammatory response in driving tumor progression, angiogenesis, and metastatic dissemination has been extensively documented [11,12,13,14]. Among various blood-based inflammatory markers, the neutrophil-to-lymphocyte ratio (NLR) has emerged as a highly cost-effective and readily available systemic index [15]. An elevated NLR reflects a dual pro-tumorigenic state: neutrophils promote a microenvironment conducive to tumor growth and extracellular matrix remodeling [16], while a concurrent reduction in lymphocytes indicates impaired cell-mediated anti-tumor immunity [17]. Although a high NLR is widely established as an independent prognostic factor for poor survival and disease recurrence in advanced CRC [11,18], its specific predictive capability regarding LNM in early T1 CRC remains less explored and occasionally controversial. Some preliminary evidence suggests that elevated pre-treatment NLR levels correlate with increased risks of nodal involvement in T1 CRC; however, robust multi-center datasets and definitive clinical cut-off values are still lacking to justify its implementation. Furthermore, while several machine learning (ML) models have been developed to predict long-term oncological outcomes, whether incorporating preoperative NLR into these models can significantly improve preoperative risk stratification beyond conventional clinicopathological variables remains entirely unclear.

Therefore, this retrospective study aims to evaluate the diagnostic performance and clinical utility of preoperative NLR in predicting LNM specifically in patients with stage T1 CRC. By analyzing the correlation between peripheral inflammatory dynamics and definitive nodal status, we seek to provide clinicians with a reliable, objective tool to improve risk stratification, potentially sparing low-risk patients from unnecessary radical surgeries while optimizing therapeutic strategies for those at true risk of metastasis.

## 2. Methods

### 2.1. Patients

This retrospective cohort study reviewed clinical data from 533 patients with stage T1 CRC who underwent surgical treatment at Sir Run Run Shaw Hospital, affiliated with Zhejiang University School of Medicine, between January 2015 and December 2024. The patients’ medical records were reviewed after approval from the Ethics Committee of Sir Run Run Shaw Hospital, which waived the requirement for written consent. All patients underwent standard surgical resection, with the diagnosis of stage pT1 CRC confirmed by postoperative histopathological evaluation.

**Figure 1:**
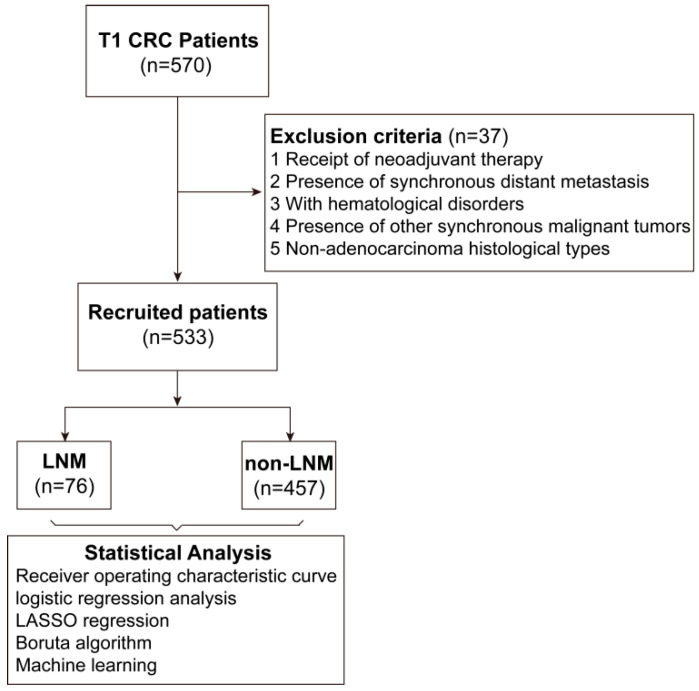
Flowchart of patient selection for the study cohort. CRC, colorectal cancer; LNM, lymph node metastasis; LASSO, Least Absolute Shrinkage and Selection Operator.

The inclusion criteria were as follows: (1) Pathologically confirmed primary colorectal adenocarcinoma; (2) Complete preoperative routine blood test results available within 2 weeks prior to surgery; (3) No history of medications or treatments known to alter hematological parameters (e.g., corticosteroids or bone marrow stimulants) within 1 month before surgery; (4) Definitive postoperative pathological confirmation of T1 stage disease. The exclusion criteria were as follows: (1) Receipt of neoadjuvant therapy, including radiotherapy, chemotherapy, immunotherapy, or targeted therapy, prior to surgery; (2) Presence of synchronous distant metastasis at the time of initial diagnosis; (3) Concurrent severe acute/chronic infection or primary hematological disorders; (4) Presence of other synchronous malignant tumors; (5) Non-adenocarcinoma histological types, such as neuroendocrine tumors or gastrointestinal stromal tumors. The flow chart of the study is shown in Figure 1.

### 2.2. Data Collection

Baseline clinical, laboratory, and pathological data were extracted from the institutional electronic medical record system. The collected variables were categorized as follows: (1) Demographic and Clinical Data: Patient age and sex; (2) Preoperative Laboratory Parameters: Complete blood count and biochemical metrics obtained within 2 weeks prior to surgery, including white blood cell (WBC) count, red blood cell (RBC) count, hemoglobin (Hb), platelet (PLT) count, absolute neutrophil (NEU) count, absolute lymphocyte (LYN) count, serum total protein (TP), albumin (Alb), high-sensitivity C-reactive protein (hsCRP), and triglyceride (TG) and serum ferritin levels; (3) Histopathological Characteristics: Features evaluated from the resected tumor specimens, including tumor differentiation (histological grade) and LVI.

### 2.3. Measurement and Calculation of NLR

NLR was selected as the primary independent variable. To minimize the confounding influence of subsequent clinical interventions, peripheral absolute neutrophil and lymphocyte counts (×10^9^/L) were obtained from the first routine blood test performed upon patient admission. The NLR was subsequently calculated as the absolute neutrophil count divided by the absolute lymphocyte count.

### 2.4. Statistical Analysis

Patients were stratified into two cohorts based on definitive postoperative pathological outcomes: the LNM group and the non-LNM group. Baseline characteristics were compared between the two groups. Continuous variables were first evaluated for normality using the Kolmogorov–Smirnov test. Normally distributed data were expressed as mean ± standard deviation (SD) and compared using the independent-samples Student’s *t*-test. Non-normally distributed variables were presented as median and interquartile range (IQR) and compared via the Mann–Whitney U test. Categorical variables were expressed as frequencies and percentages, with differences analyzed using the Pearson chi-square (χ^2^) test or Fisher’s exact test, as appropriate. Variables with statistical significance in the univariate analysis were subsequently included in the multivariate logistic regression analysis to identify independent risk factors associated with LNM. Receiver operating characteristic (ROC) curve analysis was conducted to evaluate the diagnostic performance of the preoperative NLR in predicting LNM, and the area under the curve (AUC) was calculated. The optimal cutoff value for NLR was determined using the Youden index, which was subsequently used to dichotomize NLR for risk assessment. To identify independent risk factors associated with LNM, variables demonstrating statistical significance in univariate analysis were entered into a multivariate logistic regression model. Odds ratios (ORs) and 95% confidence intervals (CIs) were calculated, and forest plots were generated to visualize the independent effects across the overall cohort and clinical subgroups. Subgroup and interaction analyses were further performed to ensure the stability of NLR as a predictor across potential confounding strata. Traditional statistical analyses and visualization were performed using SPSS software (version 27.0) and R software (version 4.4.2). All tests were two-sided, and a *p*-value < 0.05 was considered statistically significant.

### 2.5. Construction and Performance Evaluation of Machine Learning Models

The total cohort of patients was randomly partitioned into training and validation cohorts at a 7:3 ratio. To prevent multicollinearity, we evaluated correlations among the variables using Spearman rank correlation analysis. For any pairs exhibiting significant collinearity (defined as Spearman correlation > 0.6), the variable with lower clinical relevance was excluded from downstream analysis. Feature selection was performed using the intersection of variables identified by Least Absolute Shrinkage and Selection Operator (LASSO) regression and the Boruta algorithm. The Boruta algorithm was utilized to identify important features by iteratively comparing the Z-scores of original variables against randomly permuted “shadow features”. Concurrently, LASSO regression was applied to reduce dimensionality, eliminate redundant variables, and mitigate model overfitting. Only variables identified by both methods were retained for subsequent model development.

Based on the selected features, six ML models were constructed: Logistic Regression (LR), Random Forest (RF), Extreme Gradient Boosting (XGBoost), Light Gradient Boosting Machine (LightGBM), Categorical Boosting (CatBoost), and Logistic Regression with Splines (LR-Spline) [19,20,21]. To address class imbalance, class weights (class_weight or scale_pos_weight) were incorporated into both the linear and tree-based algorithms.

To ensure model stability and optimize hyperparameters, a stratified 5-fold cross-validation grid search was executed within the training cohort. In each iteration, the training dataset was partitioned into five subsets with balanced outcome distributions; four folds were used for model fitting and the remaining fold served as the internal validation set. Out-of-fold (OOF) prediction probabilities from each fold were retained to evaluate internal model discrimination, calibration, and clinical utility. The aggregated OOF-AUC was adopted as the primary metric to determine the optimal hyperparameter combinations and select the final model architecture. The final model performance was subsequently validated on the independent validation cohort.

Model performance was comprehensively evaluated using multiple metrics, including balanced accuracy, sensitivity (recall), specificity, the Brier score, and the AUC. To address the “black-box” nature of the ML algorithms and facilitate clinical translation, Shapley Additive Explanation (SHAP) values—derived from coalitional game theory—were applied to the optimal model. SHAP values were utilized to quantify and visualize both global feature importance (plotted as the mean absolute SHAP value bar chart) and individual variable contributions to specific clinical predictions. All ML workflows, including feature selection, model training, validation, and interpretability analyses, were executed using Python (version 3.12.0) with the scikit-learn, xgboost, lightgbm, catboost, and shap packages.

### 2.6. Handling of Missing Values

Variables with more than 20% missing data were excluded from the analysis. The proportion of missing values for all variables prior to imputation is presented in Supplementary Table S1. For the remaining variables, missing values were imputed using multiple imputation by chained equations (MICE) via the package in R (version 4.4.2). We generated 5 imputed datasets (m = 5) across 10 iterative cycles per chain (maxit = 10), utilizing a fixed random seed (seed = 123) to guarantee reproducibility.

## 3. Result

### 3.1. Baseline Clinical and Demographical Characteristics

A total of 533 patients with pathologically confirmed primary T1 colorectal adenocarcinoma were retrospectively evaluated (Figure 1); their baseline clinical and preoperative laboratory characteristics are summarized in Table 1. Overall, LNM was present in 76 patients (14.3%) and absent in 457 (85.7%). The median cohort age was 63 years (IQR 56–69), and 328 patients (61.5%) were male. Histological and morphological evaluations revealed that LVI was present in 63 patients (11.8%). Tumor differentiation was classified as G1 in 267 patients (50.1%), G2 in 206 (38.6%), and G3 in 38 (7.1%), while the degree of differentiation was undetermined in 22 cases (4.1%). The vast majority of tumors exhibited a protruded morphology (*n* = 508, 95.3%) and were located in the left-sided colorectum (*n* = 456, 85.6%). Comorbidities and risk factors were minimal: 482 patients (90.4%) had no history of prior malignancies, 516 (96.8%) had no family history of CRC, and preoperative anemia and hypoproteinemia were observed in only 39 (7.3%) and 31 patients (5.8%), respectively. Regarding preoperative laboratory parameters, the median NLR was 2.14 (IQR 1.59–2.88). Other key baseline systemic markers included a median hsCRP of 1.1 mg/L (IQR 0.6–2.5), median Hb of 139 g/L (IQR 127–151), and median Alb of 42.9 g/L (IQR 39.8–45.1).

### 3.2. Optimal NLR Cutoff for LNM

**Figure 2:**
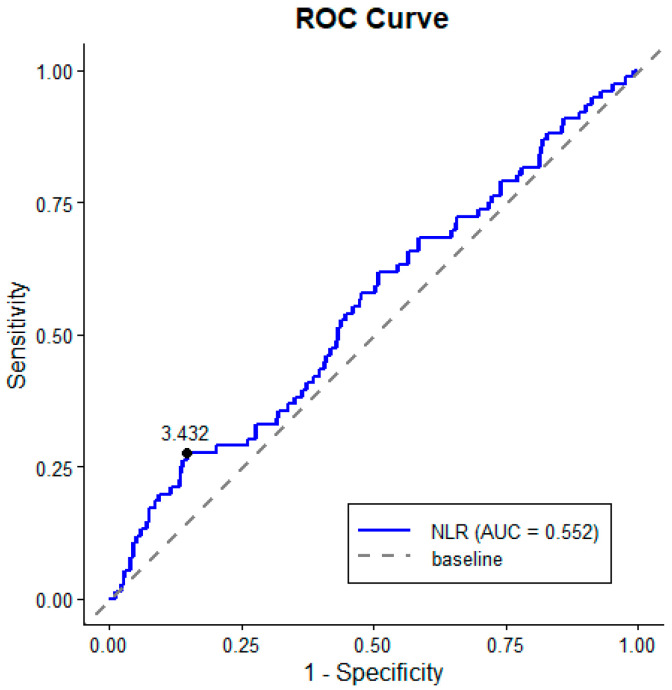
ROC curve of preoperative NLR for predicting LNM in patients with T1 CRC. The black dot represents the optimal cutoff threshold determined by the maximum Youden index. The dashed line represents the reference line, and the solid line represents the NLR performance curve. AUC, area under the curve; NLR, neutrophil-to-lymphocyte ratio; ROC, receiver operating characteristic.

The ROC curve analysis was performed to evaluate the diagnostic performance of the preoperative NLR for predicting LNM. Based on the maximum Youden index, the optimal NLR cutoff value was determined to be 3.432. At this threshold, 88 patients (16.5%) were classified into the NLR_high group (≥3.432), while 445 patients (83.5%) were in the NLR_low group (<3.432). This cutoff yielded an AUC of 0.552 (95% CI: 0.480–0.623), with a sensitivity of 27.63% and a specificity of 85.34% (Figure 2). These findings indicate that while a high NLR possesses strong specificity, NLR as a standalone biomarker has limited discriminatory power for LNM prediction, underscoring the necessity of integrating it with additional clinicopathological features in subsequent predictive modeling.

### 3.3. Univariate Analysis of Factors Associated with LNM

In the univariate analysis, the LNM group demonstrated a significantly higher prevalence of LVI, high-grade tumor differentiation (G3), depressed tumor morphology, and elevated NLR (≥3.432), as well as a greater rate of preoperative anemia and a history of prior malignancies (all *p* < 0.05). Additionally, preoperative Hb levels and RBC counts were significantly lower in patients with LNM compared to those without LNM (*p* = 0.028 and *p* = 0.045, respectively). No significant differences were observed between the two groups regarding age, sex, tumor location, or family history of CRC (all *p* > 0.05). Similarly, other preoperative laboratory parameters—including WBC, PLT, NEU, LYN, and monocyte counts, as well as TP, Alb, lipid profiles, hsCRP, and ferritin—showed no statistically significant association with LNM status (all *p* > 0.05).

### 3.4. Multivariate Analysis of LNM

**Figure 3:**
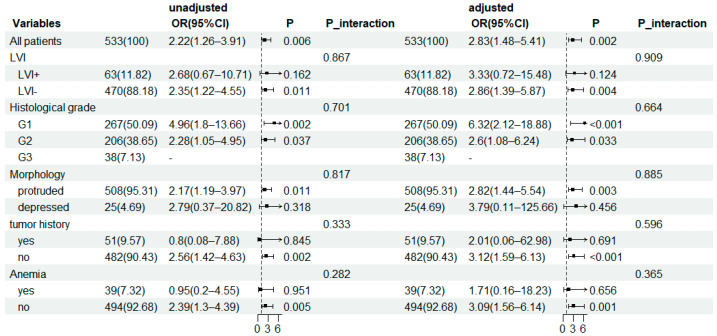
Forest plot of binary logistic regression and subgroup analysis. LVI, lymphovascular invasion; OR, odds ratio; CI, confidence interval.

To identify independent predictors of LNM in CRC, we performed univariate and multivariate binary logistic regression analyses. Potential risk factors demonstrating significant differences in Table 2—namely NLR, LVI, histological grade, tumor morphology, tumor history, and anemia—were included in the initial evaluation. Variables with a *p*-value < 0.05 in the univariate analysis were subsequently entered into the multivariate logistic regression model to adjust for potential confounders. In the overall cohort of 533 patients, high NLR was significantly associated with an increased risk of LNM in the unadjusted model (unadjusted OR = 2.22, 95% CI: 1.26–3.91, *p* = 0.006). After adjusting for LVI, histological grade, tumor morphology, tumor history, and anemia, high NLR remained a robust independent predictor of LNM (adjusted OR = 2.83, 95% CI: 1.48–5.41, *p* = 0.002) (Figure 3). To assess the consistency of this association across clinically relevant subgroups, we performed stratified logistic regression analyses. In the unadjusted models, high NLR was consistently associated with an increased risk of LNM among patients who were LVI-negative and had G1/G2 histological grade, protruded tumor morphology, no personal tumor history, and no anemia (all *p* < 0.05). Following multivariate adjustment, these associations remained statistically significant, particularly within the LVI-negative (adjusted OR = 2.86, 95% CI: 1.39–5.87, *p* = 0.004) and G1 subgroups (adjusted OR = 6.32, 95% CI: 2.12–18.88, *p* < 0.001). Notably, no significant interactions were observed between high NLR and any of the stratifying variables (all P_interaction > 0.05), confirming that the association between NLR and LNM was robust across subgroups. The G3 histological grade subgroup (*n* = 38) was excluded from effect estimation due to complete separation, which precluded reliable OR calculation. Similarly, the association did not reach statistical significance in the LVI-positive (*n* = 63), depressed morphology (*n* = 25), positive tumor history (*n* = 51), and anemia (*n* = 39) subgroups, likely due to limited sample sizes resulting in insufficient statistical power. Nevertheless, the direction of the effect estimates in these subgroups remained entirely consistent with the overall cohort (adjusted ORs: 3.33 for LVI-positive, 3.79 for depressed morphology, 2.01 for tumor history, and 1.71 for anemia), suggesting a similar underlying trend.

### 3.5. Feature Selection for Machine Learning

**Figure 4:**
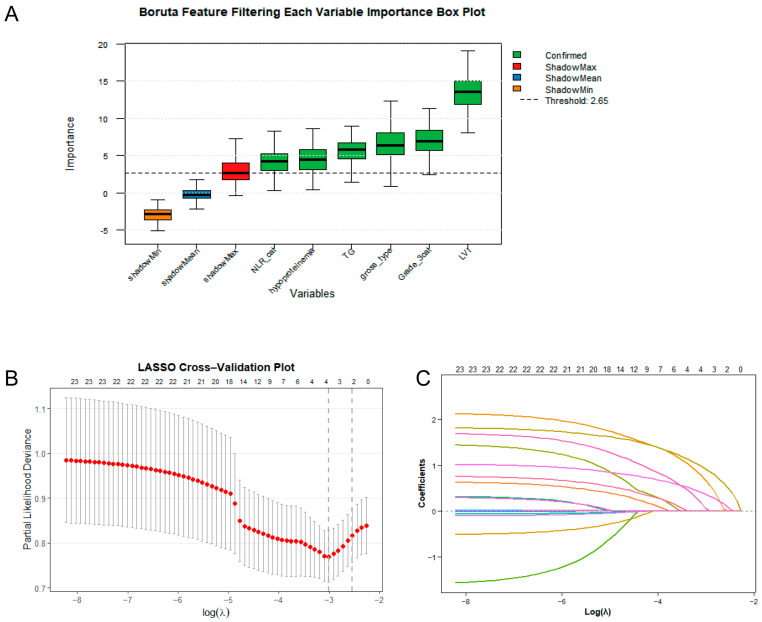
Feature selection with Boruta and LASSO regression. (**A**) Feature selection using the Boruta algorithm for predicting lymph node metastasis. The horizontal axis is the name of each variable, and the vertical axis is the Z value of each variable. The box plot shows the Z value of each variable during model calculation. (**B**) LASSO regression cross-validation plot for feature selection. (**C**) LASSO coefficient paths for feature selection of lymph node metastasis. LVI, lymphovascular invasion; TG, triglyceride; NLR, neutrophil-to-lymphocyte ratio.

The study cohort comprised 533 patients and was randomly divided into a training cohort (*n* = 374) and an independent validation cohort (*n* = 159) at a 7:3 ratio. The training cohort included 55 patients with LNM, while the validation cohort included 21 patients with LNM. To identify the most informative predictors of LNM, a dual feature-selection strategy combining the Boruta algorithm and LASSO regression was applied to the training data. The Boruta algorithm identified 6 confirmed features that exhibited importance scores significantly exceeding the shadow feature threshold: LVI, histological grade, tumor morphology, TG, hypoproteinemia, and NLR. Among these, LVI, histological grade, and tumor morphology ranked highest in terms of Boruta Z-scores (Figure 4A). Separately, LASSO regression was performed using 10-fold cross-validation to determine the optimal penalty parameter (λ = 0.0494). At this optimal λ, 4 variables with non-zero coefficients were retained: tumor morphology, LVI, histological grade, and NLR (Figure 4B,C). By taking the intersection of both feature-selection approaches, a final panel of 4 core predictive features was selected for downstream model development: tumor morphology, LVI, histological grade, and NLR.

### 3.6. Comparative Performance of Machine Learning Models

**Figure 5:**
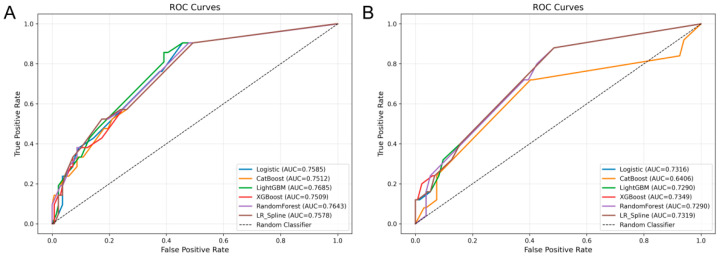
ROC curves of the machine learning models for predicting lymph node metastasis. (**A**) Models incorporating NLR. (**B**) Models excluding NLR. AUC, area under the curve; CatBoost, Categorical Boosting; LightGBM, Light Gradient Boosting Machine; LR-Spline, logistic regression with splines; OOF, out-of-fold; XGBoost, Extreme Gradient Boosting.

ROC curves were constructed to assess the discriminative ability of each model for predicting LNM in patients with T1 CRC (Figure 5A). Among the six ML models, LightGBM achieved the highest AUC at 0.768 (95% CI: 0.666–0.860). In comparison, the AUCs for LR, LR-Spline, RF, CatBoost, and XGBoost were 0.758 (95% CI: 0.648–0.862), 0.758 (95% CI: 0.641–0.859), 0.764 (95% CI: 0.651–0.866), 0.751 (95% CI: 0.638–0.853), and 0.751 (95% CI: 0.632–0.853), respectively (Table 3). Beyond discrimination, the models were evaluated based on accuracy, recall, specificity, and Brier score (Table 3). LightGBM demonstrated the best overall predictive performance and favorable calibration, yielding a balanced_accuracy of 0.7329, a specificity of 0.6086, a recall of 0.8571, and a Brier score of 0.105. Consequently, LightGBM was selected as the optimal model for subsequent interpretation and validation analyses.

To evaluate the incremental predictive value of preoperative NLR, a baseline LightGBM model comprising only conventional clinicopathological predictors (tumor morphology, LVI, and histological grade) (Figure 5B) was compared against the expanded model incorporating NLR. Within the independent validation cohort, the inclusion of NLR improved the validation AUC from 0.729 to 0.768, demonstrating that preoperative NLR provides meaningful complementary predictive value beyond established pathological risk factors.

### 3.7. SHAP-Based Model Interpretation

**Figure 6:**
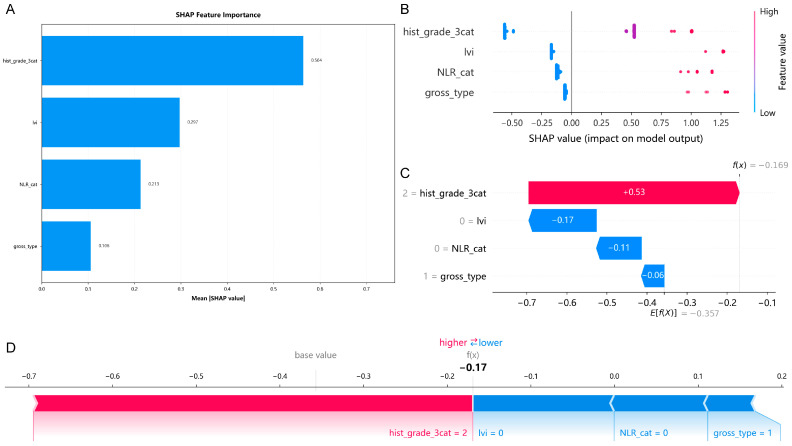
SHAP explanation of the LightGBM model for predicting lymph node metastasis. (**A**) SHAP summary bar plot displaying the global feature importance ranking. (**B**) SHAP summary dot plot illustrating the impact of each feature on the model output; each dot represents an individual patient, with color indicating the feature value (red: high; blue: low). Wider horizontal dispersion indicates a greater impact on the model’s prediction. (**C**) SHAP waterfall plot showing the contribution of each feature to the prediction result of a representative patient. Red bars indicate features that contribute positively to the prediction, while blue bars indicate negative contributions. (**D**) SHAP force plot showing a personalized prediction for an individual patient. Red bars denote risk factors pulling the prediction higher, while blue bars represent protective factors, with the length of each bar corresponding to the magnitude of the effect. LVI, lymphovascular invasion; SHAP, Shapley Additive Explanations; NLR, neutrophil-to-lymphocyte ratio.

The SHAP method was applied to evaluate the contribution of each predictive feature, providing interpretable insights at both the global and individual patient levels (Figure 6). At the global level, the mean absolute SHAP value was used to determine overall feature importance (Figure 6A). Histological grade was identified as the most influential predictor (mean SHAP = 0.564), followed by LVI, NLR, and tumor morphology. The SHAP summary plot from the validation cohort (Figure 6B) illustrates the direction and magnitude of these features’ effects, where red represents high feature values and blue represents low values. Specifically, an increased risk of LNM was associated with a higher histological grade, the presence of LVI, elevated NLR, and depressed tumor morphology. For individual-level interpretation, SHAP values for a representative patient without LNM are presented in Figure 6C. This patient presented with a G2 histological grade, negative LVI, a low NLR and a protruding tumor morphology. The patient’s overall SHAP value was −0.169, indicating a low predicted risk of LNM. Overall, the SHAP analysis provided transparent, consistent explanations of model behavior, validating the clinical relevance of these features while precisely quantifying their individual contributions to LNM risk stratification.

## 4. Discussion

Accurate preoperative prediction of LNM is critical for tailoring individualized surgical strategies and prognostic evaluations in CRC. In this study, we demonstrated that an elevated preoperative NLR was independently associated with an increased risk of LNM, even after adjustment for relevant clinicopathological factors. Although NLR alone exhibited limited standalone discriminatory ability, its integration into a multivariable ML framework improved predictive performance beyond conventional clinicopathological variables. These findings suggest that NLR should not be viewed in isolation, but rather as a complementary biomarker of systemic inflammation that enhances individualized risk stratification when combined with established pathological predictors. The present study offers several distinct contributions to the existing literature. First, it focuses specifically on T1 CRC, a clinical scenario where precise preoperative assessment of LNM is vital for deciding between local excision and radical surgery. Second, unlike previous studies that primarily evaluated the prognostic significance of NLR, we investigated its value as a preoperative predictor of LNM. Third, we demonstrated that incorporating NLR into ML models improved predictive performance beyond conventional clinicopathological variables while maintaining model interpretability through SHAP analysis.

The biological association between systemic inflammation and tumor dissemination may explain the predictive contribution of NLR. An elevated NLR typically reflects a systemic inflammatory state characterized by neutrophil-mediated tumor promotion and suppressed lymphocyte-mediated anti-tumor immunity [16,17]. Neutrophil-derived inflammatory cytokines facilitate tumor angiogenesis, extracellular matrix remodeling, and epithelial–mesenchymal transition (EMT) [22], thereby enhancing tumor invasive and metastatic potential. Conversely, impaired lymphocyte function attenuates immune surveillance against tumor progression, collectively creating a pro-metastatic tumor microenvironment that facilitates lymphatic dissemination. Compared with traditional serum tumor markers such as CEA and CA19-9, NLR is derived from routine preoperative blood tests without additional financial or procedural costs, conferring superior clinical accessibility. Furthermore, subgroup analysis verified the stability and universality of the NLR-LNM association across major clinicopathological subgroups, suggesting that its predictive value is not substantially affected by conventional clinical confounders. While several small-sample subgroups exhibited insufficient statistical power or data separation due to limited case numbers, these findings warrant cautious interpretation and further validation in larger multicenter cohorts.

Through a dual-feature selection procedure combining LASSO regression and the Boruta algorithm, we identified a core panel of predictive variables for LNM in T1 CRC. Among the evaluated algorithms, the LightGBM model demonstrated favorable discrimination and calibration performance on the independent validation cohort. Compared with traditional regression approaches that primarily capture linear associations, ML methods are capable of modeling complex nonlinear relationships and high-order interactions among clinical variables. Furthermore, SHAP analysis improved model interpretability by demonstrating that established pathological factors, particularly histological grade and LVI, remained major contributors to LNM prediction, while systemic inflammatory indicators, such as NLR, provided vital complementary predictive value. These findings support the biological plausibility and clinical interpretability of the proposed model.

The clinical significance of this study lies in extending the utility of NLR from a prognostic marker mainly investigated in advanced CRC to a preoperative risk stratification tool for early-stage T1 CRC. Currently, treatment decisions following ER rely primarily on classical pathological risk factors, such as LVI and poor histological differentiation. Our findings suggest that integrating NLR with these routine clinicopathological variables provides a more refined, individualized risk estimation than conventional factors alone. This approach could complement existing guideline-based assessments, helping clinicians identify high-risk patients who warrant additional radical surgery while sparing low-risk individuals from unnecessary, morbidity-associated procedures. However, prospective studies directly comparing ML-based prediction models with current clinical guideline criteria are essential before widespread clinical implementation.

Adequate lymph node evaluation is critical for the reliable classification of nodal status. In our cohort, the median number of examined lymph nodes was 15, with approximately 78.6% of patients meeting the guideline-recommended threshold of at least 12 lymph nodes. Notably, the proportion of patients meeting this threshold was comparable between the LNM and non-LNM groups, suggesting that variation in lymph node harvest was unlikely to introduce systematic stage migration bias. Nevertheless, the possibility of residual understaging cannot be entirely ruled out. Additionally, several guideline-recommended pathological predictors, such as tumor budding and precise submucosal invasion depth, were unavailable due to the retrospective nature of this cohort. During the earlier phase of our study period, standardized reporting of these variables was not routinely practiced, and fragmented endoscopic specimens frequently precluded reliable pathological assessment. Consequently, these missing variables reflect historical pathology practices rather than selective reporting or exclusion. Future prospective studies utilizing standardized pathological protocols are warranted to determine whether NLR retains independent, incremental predictive value when integrated with a complete set of modern guideline-recommended risk factors.

Several limitations of this study should be acknowledged. First, this was a retrospective single-center study, meaning that inherent selection bias cannot be entirely excluded. Second, although internal validation was performed using a held-out validation cohort, external validation in diverse, multicenter populations remains essential to confirm the generalizability of our model. Third, despite utilizing robust dimensionality reduction through feature selection, the relatively limited number of LNM events may still introduce a risk of model overfitting or optimism. Fourth, peripheral NLR can be influenced by transient systemic inflammatory conditions, subclinical infections, or unmeasured medications, potentially resulting in residual confounding. Finally, the optimal NLR threshold was derived directly from the current dataset; thus, this population-specific cutoff requires external validation in independent cohorts before it can be applied in routine clinical practice.

In conclusion, an elevated preoperative NLR is associated with an increased risk of LNM in patients with T1 CRC. By integrating the NLR with selected clinicopathological variables, our ML framework provides improved risk stratification compared with conventional staging approaches alone. Although further external validation is required, this study highlights the potential value of combining systemic inflammatory biomarkers with interpretable ML methods to guide individualized management decisions following the ER of early CRC.

## 5. Conclusions

This study demonstrated that an elevated preoperative NLR is independently associated with LNM in T1 CRC patients. Although NLR alone exhibits limited standalone discriminatory power, it provides valuable complementary predictive value when integrated with routine clinicopathological variables. By leveraging these factors, we developed and internally validated multiple ML models; among these, the optimized LightGBM model demonstrated favorable discrimination and potential clinical utility. These findings suggest that incorporating preoperative NLR into a multivariable ML framework can improve individualized risk stratification, helping guide the critical decision between active surveillance after ER and proceeding to additional radical surgery.

## Figures and Tables

**Table 1:** Baseline clinical and laboratory characteristics of the study cohort with T1 colorectal cancer.

Variable	*N* (%)	Variable	Median (IQR)/Mean (±SD)
Sex		Age	63 (56, 69)
Female	205 (38.5)	RBC	4.5 (±0.5)
Male	328 (61.5)	PLT	206 (170, 247)
LNM		WBC	5.8 (4.9, 6.9)
no	457 (85.7)	NEU	3.5 (2.81, 4.455)
yes	76 (14.3)	LYN	1.64 (1.315, 2.025)
LVI		NLR	2.14 (1.59, 2.88)
negative	470 (88.2)	Hb	139 (127, 151)
positive	63 (11.8)	monocyte	0.35 (0.28, 0.42)
Histological grade		TP	70.9 (66.5, 74.6)
G1	267 (50.1)	Alb	42.9 (39.8, 45.1)
G2	206 (38.6)	TG	1.4 (1.01, 1.92)
G3	38 (7.1)	cholesterol	4.89 (4.18, 5.65)
missing	22 (4.1)	hsCRP	1.1 (0.6, 2.5)
Morphology		Ferritin	202.8 (110.15, 349.75)
protrude	508 (95.3)	Number of examined lymph nodes	15 (12, 19)
depressed	25 (4.7)		
Location			
left	456 (85.6)		
right	77 (14.4)		
Other tumor history			
no	482 (90.4)		
yes	51 (9.6)		
Family history			
no	516 (96.8)		
yes	17 (3.2)		
Anemia			
no	494 (92.7)		
yes	39 (7.3)		
Hypoproteinemia			
no	502 (94.2)		
yes	31 (5.8)		
Adequate lymph node harvest (≥12 nodes)	419 (78.6)		

Note: Alb, albumin; Hb, hemoglobin; hsCRP, high-sensitivity C-reactive protein; LNM, lymph node metastasis; LVI, lymphovascular invasion; LYN, lymphocyte; NEU, neutrophil; NLR, neutrophil-to-lymphocyte ratio; PLT, platelet; RBC, red blood cell; TP, total protein; TG, triglyceride; WBC, white blood cell.

**Table 2:** Univariate analysis of clinicopathological factors associated with LNM in T1 colorectal cancer.

	LNM (*n* = 76)	non-LNM (*n* = 457)	χ^2^/t/Z	*p*
Age, years	63 (58, 69)	63 (56, 69.5)	−0.36	0.719
Male, *n* (%)	51 (67.1)	277 (60.6)	1.161	0.281
LVI (+)	25 (32.9)	38 (8.3)	37.772	<0.001
Histological grade (Por or Muc)	13 (17.8)	25 (5.7)	33.354	<0.001
Morphology (depressed)	10 (13.2)	15 (3.3)	24.216	<0.001
NLR_high	21 (27.6)	67 (14.7)	7.953	0.005
Location (left)	68 (89.5)	388 (84.9)	1.102	0.294
Tumor history (yes)	12 (15.8)	39 (8.5)	3.964	0.046
Family history (yes)	1 (1.3)	16 (3.5)	0.424	0.515
Anemia (yes)	10 (13.2)	29 (6.3)	4.459	0.035
WBC	5.95 (4.9, 7.15)	5.8 (4.9, 6.9)	−0.554	0.58
RBC	4.4 ± 0.5	4.5 ± 0.5	−2.007	0.045
Hb	133 (126, 148.75)	140 (128, 151)	−2.204	0.028
PLT	203.5 (161.25, 254.75)	207 (171, 246)	−0.13	0.897
NEU	3.58 (2.88, 4.712)	3.49 (2.795, 4.42)	−0.697	0.486
LYN	1.575 (1.2775, 1.85)	1.64 (1.32, 2.04)	−1.616	0.106
monocyte	0.355 (0.28, 0.465)	0.35 (0.28, 0.42)	−0.166	0.868
TP	70.4 (65.9, 75)	71.1 (66.6, 74.6)	−0.532	0.595
Alb	41.9 (38.1, 45.1)	43 (39.9, 45.15)	−1.037	0.3
TG	1.4 (1.08, 1.88)	1.39 (0.99, 1.92)	−0.102	0.919
cholesterol	4.9 (4.11, 5.66)	4.89 (4.2, 5.65)	−0.261	0.794
hsCRP	1.1 (0.6, 1.9)	1.1 (0.6, 2.7)	−0.655	0.513
Ferritin	170.05 (101.33, 367.05)	204.7 (113.9, 347.5)	−0.416	0.678
Hypoproteinemia (yes)	6 (7.9)	25 (5.5)	0.327	0.568
Number of examined lymph nodes	16 (12, 20)	15 (12, 19)	−0.467	0.641
Adequate lymph node harvest (≥12 nodes)	58 (76.3)	361 (79)	0.278	0.598

Note: Alb, albumin; Hb, hemoglobin; hsCRP, high-sensitivity C-reactive protein; LNM, lymph node metastasis; LVI, lymphovascular invasion; LYN, lymphocyte; NEU, neutrophil; NLR, neutrophil-to-lymphocyte ratio; PLT, platelet; RBC, red blood cell; TP, total protein; TG, triglyceride; WBC, white blood cell.

**Table 3:** Comparison of model performance metrics for predicting lymph node metastasis.

Model	AUC (95% CI)	Balanced_Accuracy	Recall (Sensitivity)	Specificity	Brier Score
Logistic	0.758 (0.648–0.862)	0.7241	0.9047	0.5434	0.2458
LR_Spline	0.758 (0.641–0.859)	0.7060	0.9047	0.5072	0.1903
CatBoost	0.751 (0.638–0.853)	0.7060	0.9047	0.5072	0.1833
LightGBM	0.768 (0.666–0.860)	0.7329	0.8571	0.6086	0.105
XGBoost	0.751 (0.632–0.853)	0.7060	0.9047	0.5072	0.1845
RandomForest	0.764 (0.651–0.866)	0.7132	0.9047	0.5217	0.1794

Note: AUC, area under the curve; CatBoost, Categorical Boosting; LightGBM, Light Gradient Boosting Machine; LR-Spline, logistic regression with splines; XGBoost, Extreme Gradient Boosting.

## Data Availability

The data presented in this study are available on request from the corresponding author due to patient privacy protection requirements, institutional review board regulations, and hospital medical record management restrictions.

## Supplementary Materials

The following supporting information can be downloaded at: https://www.mdpi.com/article/10.3390/cancers18152370/s1, Table S1: Missing data distribution before imputation.
