# Supplementary material for: Integration of Preoperative Neutrophil-to-Lymphocyte Ratio into Machine Learning Models for Predicting Lymph Node Metastasis in CRC
Source: Cancers (Basel). 2026 Jul 23;18(15):2370. doi: 10.3390/cancers18152370 (PMC13464815; doi:10.3390/cancers18152370)
Supplement: Supplementary file 1 [file cancers-18-02370-s001.zip › cancers-4413922-supplementary.pdf]

Supplementary Table S1. Missing data distribution before imputation

Variable	n_missing	pct_missing(%)
Ferritin	36	6.75422138836773
TG	29	5.44090056285178
cholestorel	29	5.44090056285178
CA125	29	5.44090056285178
CK	28	5.25328330206379
CA199	19	3.56472795497186
AFP	19	3.56472795497186
CEA	18	3.37711069418387
TP	5	0.938086303939963
Alb	5	0.938086303939963
hsCRP	5	0.938086303939963
hypoproteinemia	5	0.938086303939963

Note: AFP,Alpha-fetoprotein; Alb, albumin; CEA, carcinoembryonic antigen; CK, creatine kinase; hsCRP, high-sensitivity C-reactive protein; TP, total protein; TG, triglyceride.
